# ATAD3A mediates activation of RAS-independent mitochondrial ERK1/2 signaling, favoring head and neck cancer development

**DOI:** 10.1186/s13046-022-02274-9

**Published:** 2022-01-29

**Authors:** Liwei Lang, Reid Loveless, Juan Dou, Tiffany Lam, Alex Chen, Fang Wang, Li Sun, Jakeline Juarez, Zhaohui Steve Qin, Nabil F. Saba, Chloe Shay, Yong Teng

**Affiliations:** 1 Department of Oral Biology and Diagnostic Sciences, Georgia Cancer Center, Augusta University, Augusta, GA 30912 USA; 2 Department of Hematology and Medical Oncology, Winship Cancer Institute, Emory University School of Medicine, 201 Dowman Dr, Atlanta, GA 30322 USA; 3 Department of Biostatistics and Bioinformatics, Emory University, Atlanta, GA 30322 USA; 4 Wallace H. Coulter Department of Biomedical Engineering, Georgia Institute of Technology and Emory, University, Atlanta, GA 30322 USA

**Keywords:** ATAD3A, HNSCC, Mitochondrial ERK1/2, WA dead mutant, VDAC1, RAS

## Abstract

**Background:**

Targeting mitochondrial oncoproteins presents a new concept in the development of effective cancer therapeutics. ATAD3A is a nuclear-encoded mitochondrial enzyme contributing to mitochondrial dynamics, cholesterol metabolism, and signal transduction. However, its impact and underlying regulatory mechanisms in cancers remain ill-defined.

**Methods:**

We used head and neck squamous cell carcinoma (HNSCC) as a research platform and achieved gene depletion by lentiviral shRNA and CRISPR/Cas9. Molecular alterations were examined by RNA-sequencing, phospho-kinase profiling, Western blotting, RT-qPCR, immunohistochemistry, and immunoprecipitation. Cancer cell growth was assessed by MTT, colony formation, soft agar, and 3D cultures. The therapeutic efficacy in tumor development was evaluated in orthotopic tongue tumor NSG mice.

**Results:**

ATAD3A is highly expressed in HNSCC tissues and cell lines. Loss of ATAD3A expression suppresses HNSCC cell growth and elicits tumor regression in orthotopic tumor-bearing mice, whereas gain of ATAD3A expression produces the opposite effects. From a mechanistic perspective, the tumor suppression induced by the overexpression of the Walker A dead mutant of ATAD3A (K358) produces a potent dominant-negative effect due to defective ATP-binding. Moreover, ATAD3A binds to ERK1/2 in the mitochondria of HNSCC cells in the presence of VDAC1, and this interaction is essential for the activation of mitochondrial ERK1/2 signaling. Most importantly, the ATAD3A-ERK1/2 signaling axis drives HNSCC development in a RAS-independent fashion and, thus, tumor suppression is more effectively achieved when ATAD3A knockout is combined with RAS inhibitor treatment.

**Conclusions:**

These findings highlight the novel function of ATAD3A in regulating mitochondrial ERK1/2 activation that favors HNSCC development. Combined targeting of ATAD3A and RAS signaling may potentiate anticancer activity for HNSCC therapeutics.

**Supplementary Information:**

The online version contains supplementary material available at 10.1186/s13046-022-02274-9.

## Background

Mitochondria are highly responsive and dynamic organelles that are fundamental to energy production, cell metabolism, survival, and death. Recent evidence has also indicated that mitochondria are highly associated with cell biological behaviors that facilitate cancer hallmark capabilities [1, 2]. While non-specific anti-mitochondrial targeting may be effective against cancer cells, it is likely to produce ill effects on the growth and survival of normal cells as well. As a result, promising treatments that precisely target the specific mitochondrial proteins involved in tumor development and progression are greatly needed. It is clear, however, that understanding these cancer-associated mitochondrial proteins is the first step in the development of mitochondria-based anticancer regimens.

The ATPase family AAA-domain containing protein 3A (ATAD3A), a nuclear-encoded mitochondrial enzyme, is anchored to the mitochondrial inner membrane (MIM) with its N-terminus in contact with the mitochondrial outer membrane (MOM) [1, 3–5]. This protein contains two coiled-coil domains (CC1 and CC2), Walker A (WA) and Walker B (WB) motifs, among which, the WA motif is responsible for ATP binding in the AAA module of ATAD3A [1, 6]. Disease-causing mutations in ATAD3A often include point mutations in these motifs [1, 6]. For example, a structural in silico analysis of a WA mutant suggests that the substitution of lysine 358 to alanine (K358A) changes the affinity of the substrate-binding site for ATP and results in reduced ATPase activity of ATAD3A, which has been associated with neurodevelopmental disorders [6, 7]. As a mitochondrial protein, ATAD3A controls a broad spectrum of physiological and pathological responses with functions implicated in mitochondrial dynamics, nucleoid organization, signaling transduction, and cholesterol metabolism [5, 8, 9]. Of particular relevance to the essential processes underlying mitochondrial biogenesis, metabolism and mitophagy, ATAD3A has been considered one of most common genes linked to mitochondrial diseases in childhood, and deletion of ATAD3A and its orthologues in flies, worms, and mammals leads to embryonic lethality with growth retardation and aberrant mitochondrial activity [1, 9–11].

Increasing evidence suggests that ATAD3A is engaged in cancer development, progression, and treatment. In lung adenocarcinoma, ATAD3A overexpression correlates with tumor stage and lymphovascular involvement in patients, and silencing its expression increases mitochondrial fragmentation and cisplatin sensitivity [12]. In prostate cancer, loss of ATAD3A reduces the secretion of prostate-specific antigen and resensitizes cisplatin-resistant tumor cells to cisplatin [13]. Noticeably, ATAD3A acts as a broker of a mitochondria-endoplasmic reticulum (ER) connection to manipulate cell signaling [1, 3]. This is supported by one of our studies that demonstrated ATAD3A interacts with the WAS protein family member 3 (WASF3) metastasis-promoting protein and promotes invasion of breast and colon cancer cells through regulating GPR78-mediated stabilization of WASF3 on the boundary of the mitochondrial and ER membranes [3]. Nevertheless, a comprehensive understanding of the role ATAD3A plays in cancer development needs to be further determined.

In the present study, we focused primarily on the functional characterization of ATAD3A in head and neck squamous cell carcinoma (HNSCC) and report for the first time that ATAD3A serves as a mitochondrial oncoprotein to drive the development of this deadly disease. In HNSCC cells, the function of ATAD3A is dependent on its ATP-binding ability and is enabled by a novel regulatory mechanism in which it activates mitochondrial ERK1/2 via protein-protein interaction. Notably, the voltage-dependent anion channel 1 (VDAC1), another gatekeeper that fuels mitochondrial functions, is essential for the ATAD3A-ERK1/2 signaling, which is RAS-independent. These novel findings signify that the impairment of ATAD3A-mediated mitochondrial oncogenic signaling could be a potential therapeutic approach, which may further combine with RAS inhibition to achieve synergistic anti-HNSCC efficacy.

## Materials and methods

### Cell lines and standard assays

The human HNSCC cell lines HN6, HN8, HN12, and HN17 were gifted from Dr. W. Andrew Yeudall [14] and maintained in our lab. All cell lines were cultured in DMEM/F12 medium (50/50) containing 10% fetal bovine serum at 37 °C in a humidified incubator maintained at 5% CO_2_. Human telomerase-immortalized tonsillar keratinocytes hTERT HAK Clone 41 were a gift from Dr. A. Klingelhutz and Dr. J. Lee (University of Iowa, Iowa City, IA) and cultured in KSFM with 0.2 ng/ml EGF and 30 μg/ml bovine pituitary extract [15]. Cells with a passage number less than ten were used for the experiments in this study. Cell culture, transfection and infection, cell proliferation, immunofluorescence (IF), Western blotting, Matrigel invasion assays and colony formation assays were carried out as we previously described [16–18]. Densitometric analysis of Western blots was performed using with ImageJ Fiji (version 1.2; WS Rasband, National Institute of Health, Bethesda, MD).

### Antibodies and reagents

Antibodies that recognize ERK1/2, p-ERK1/2, RAF1, p-RAF1, AKT, p-AKT, MEK1/2, p-MEK1/2, GAPDH, COXIV, VDAC1, cleaved caspase-3, calnexin, PCNA, E-cadherin and vimentin were purchased from Cell Signaling Technology (Beverly, MA). Antibodies against β-actin, Myc, and Flag were obtained from Sigma-Aldrich (St Louis, MO). Antibodies that recognize cyclin D1 and CDK4 were purchased from Santa Cruz Biotechnology (Dallas, TX). ATAD3A antibody was purchased from Novus Biologicals (Abingdon, UK). CellTiter 96® AQueous One Solution Cell Proliferation Assay (MTS) was obtained from Promega (Madison, WI). Texas-red phalloidin, D-luciferin bioluminescent substrate, and alamarBlue Cell Viability Reagent were purchased from ThermoFisher Scientific (Waltham, MA). The RAS inhibitor salirasib and the ERK1/2 inhibitor SCH772984 were obtained from SelleckChem (Houston, TX), and 4-nitroquinoline-1-oxide (4NQO) was purchased from Sigma-Aldrich (St. Louis, MO).

### Constructs and gene modifications

To construct the Flag-tagged full-length ATAD3A gene and its WA dead mutant K358A (with the substitution of lysine 358 to alanine), DNA fragments were amplified by PCR from pcDNA5-ATAD3A and pcDNA5-ATAD3A-K358A plasmids [6] and subcloned into the lentiviral vector pCDH-CMV-MCS-EF1-PURO (System Biosciences) using XbaI and BamHI individually. An epitope Flag tag was introduced by PCR, and the primer sequences used in this assay were 5′-CGTCTAGAGCCACCATGTCGTGGCTCTTCGGC-3′ (forward primer) and 5′-ACGGATCCTTATCACTTGTCGTCATCGTCTTTGTAGTCGGATGGGGAGGGCTCGTC-3′ (reverse primer). Myc-tagged pcDNA3.1-ATAD3A-N-terminus (ATAD3A-Cter, 1-287 aa) and pcDNA3.1-ATAD3A-C-terminus (ATAD3A-Nter, 258-586 aa) plasmids were kindly provided by Dr. Rousseau Denis [19, 20]. For ATAD3A knockout (KO), a single guide RNA (sgRNA) of 20 nucleotides was designed to target a region spanning the exon 6 (5′-CCGCCGGCTCGTGGCAGTCT-3′). Guide sequences were cloned into the sgRNA scaffold of pSpCas9n (BB)-2A-Puro (PX459) V2.0 (Addgene plasmid #62988) [21]. For the isolation of ATAD3A KO clones, cells were selected by 1.0 μg/ml puromycin for 3 days after transfection, and single cells were then seeded into a 96-well plate. Derived clones were screened by Western blotting and verified by sequencing. For gene knockdown, the pLKO.1-puro TRC control shRNA targeting the green fluorescent protein (shGFP) and shRNAs targeting ATAD3A, VDAC1, and RAF1 were obtained from Horizon Discovery (Waterbeach, UK). The lentivirus generated by co-transfecting the lentiviral shRNA and packaging plasmids (Viropower kit; Invitrogen) into HEK293FT cells (Invitrogen) was used to infect HNSCC cells as previously described [16, 22].

### Soft agar colony formation and three-dimensional (3D) cell cultures

This assay was performed using a two-layer soft agar system as previously described [22]. Briefly, 2 × 10^4^ ATAD3A KO and parental HN12 cells suspended in 1.5 ml of top agar (0.3% agar in two-fold complete cell culture medium) were overlaid onto a 1.5 ml layer of bottom agar (0.6% agar in the same culture medium). After 14 days of incubation, the colonies were photographed and scored by inverted phase-contrast microscopy without fixation and staining. As for 3D culture, ATAD3A gene modified HN12 cells and their corresponding control cells (1 × 10^5^) were seeded into SeedEZ scaffolds (Lena Bioscience, Atlanta, GA) supplied with complete medium. On Day 14 after treatment, cell growth in SeedEZ scaffolds was stained by Texas-red phalloidin and measured by alamarBlue at 545/590 nm ex/em [23].

### Cell cycle analysis

Cell cycle progression was assessed by flow cytometry. Briefly, ATAD3A KO and parental HN12 cells at a density of 1 × 10^6^/ml were collected, fixed in 1% methanol-free formaldehyde (Sigma-Aldrich) for 20 min and suspended in 70% ethanol solution to dehydrate at − 20 °C for 24 h. Cells were washed and treated with 0.5μg/ml RNAse A for 10 min at room temperature, followed by incubation of 20 μg/ml propidium iodide (Invitrogen) overnight at 4 °C. Samples were analyzed using a FACSCalibur system and ModFitLT V3.2.1 software (BD Bioscience, San Jose, CA).

### Mitochondria isolation, purification and trypsin proteolysis

Mitochondrial fractions were isolated using the ProteoExtract Mitochondria/Cytosol Fractionation Kit (Millipore, Billerica, MA, USA) according to the manufacturer’s instructions. The crude mitochondria were purified from sucrose-density gradient as previously described [24]. The highly purified mitochondrial and cytoplasmic fractions were confirmed by Western blotting analysis using the mitochondrial marker COXIV, ER marker calnexin, cytoplasmic GAPDH protein and nuclear PCNA protein. For trypsin proteolysis, isolated mitochondria were treated with 50 μg/ml trypsin for 30 min and then pelleted and lysed as described previously [3].

### Immunoprecipitation (IP)

In vitro protein-protein interactions were assessed by IP as previously described [3, 25, 26]. Briefly, the cell lysate was diluted in 500 μl IP lysis buffer with the corresponding antibodies, followed by incubation with Protein A/G Sepharose® (Abcam, Cambridge, MA) for at least 4 h at 4 °C. The immunoprecipitated proteins were washed three times and then subjected to Western blotting analysis.

### Tissue microarrays and immunohistochemistry (IHC)

The human head and neck tissue microarrays (HN802 and HN803c) were purchased from US Biomax (Rockville, MD). IHC of the human tissue microarrays and paraffin-embedded xenografts was conducted as previously described [16]. Human tissue microarrays were immunostained with anti-ATAD3A antibody, and sections of tongue tumor xenografts were immunostained with antibodies against phospho-ERK1/2 and Ki67. Negative controls included non-specific polyclonal rabbit antibody at 2 μg/ml (Abcam, Cambridge, MA). Immuno-reactivity was visualized using the DAB Kit (Vector Laboratories, Burlingame, CA) and counterstained with hematoxylin according to the manufacturers’ procedure. Five areas of each sample from xenograft sections or tissue microarrays were imaged at random by a CCD camera (Zeiss), and IHC staining intensity of targeted protein was quantified using Image pro-Plus6.0 software (Media Cybernetics, Silver Springs, MD) and presented as integrated optical density (IOD).

### Transmission electron microscopy (TEM)

A standardized method was used according to our established protocol [14, 18]. Briefly, 1.0 × 10^7^ ATAD3A KO and parental HN12 cells were harvested and fixed in 2% glutaraldehyde for 45 min. The samples were postfixed in 2% osmium tetroxide for 2 h, dehydrated through a graded series of ethanol (50, 70, 90, and 100% for 15 min and then three times at 100%), and embedded in Epon-Araldite resin. Ultrathin sections were double-stained with 1% lead citrate and 0.5% uranyl acetate and examined with the JEOL JEM-1230 TEM.

### Seahorse bioanalyzer

Live cell analyses of oxygen consumption rate (OCR) and extracellular acidification rate (ECAR) were measured by Seahorse XF Cell Energy Phenotype Test Kit on a Seahorse XFe96 Extracellular Flux analyzer (Agilent Technology) as previously described [27]. In brief, 4 × 10^3^ cancer cells were seeded on each well of poly-D-lysine coated XF96 miniplates overnight and then incubated in 100 μl XF medium (10 mM glucose, 2 mM L-glutamine, and 1 mM sodium pyruvate) for 45 mins. Three measurements were obtained under basal conditions and upon sequential injection of 1 μM oligomycin (Oligo) and 1 μM fluoro-carbonyl-cyanide phenylhydrazone (FCCP). OCR and ECAR values were calculated from 3-min measurement cycles and adjusted to cell numbers. OCR:ECAR ratio was calculated according to previous report [28].

### RNA-sequencing (RNA-seq) and data analysis

Total RNA was extracted from ATAD3A KO and parental HN12 cells using TRIzol reagent (Invitrogen, Carlsbad, CA) according to the manufacturer’s protocol. The purified RNA samples were sent to Novogene Corporation (Sacramento, CA) for library construction and sequencing using the Illumina HiSeq™ 2000 platform to obtain expression libraries of 50-nt read length. Independent duplicate cultures were sampled to avoid random error. Differentially expressed genes (DEGs) were identified using the DESeq R package functions estimateSizeFactors and nbinomTest. *P* value < 0.05 and fold change > 1.25 or fold change < 0.75 was set as the threshold for significantly differential expression. Hierarchical cluster analysis of DEGs was performed to explore transcript expression patterns, and Gene Ontology (GO) was performed to identify the potential function of all DEGs. The detailed RNA-seq information of this assay is available in GSE163939 deposited in the NIH Gene Expression Omnibus (GEO) database.

### Reverse transcription quantitative PCR (RT-qPCR) and phospho-kinase profiling

RT-qPCR was performed using Applied Biosystems™ Power SYBR™ Green PCR Master Mix (ThermoFisher Scientific) on the StepOne Plus Real-Time PCR System (Applied Biosystems, Foster City, CA). Technical triplicates of each sample were examined, and gene expression levels were normalized against β-actin. In this assay, the primers for human TNFα were: 5′-TGCACTTTGGAGTGATCGGC-3′ (forward) and 5′-CTCAGCTTGAGGGTTTGCTAC-3′ (reverse). The primers for human β-actin were: 5′-GAGCACAGAGCCTCGCCTTT-3′ (forward) and 5′-TCATCATCCATGGTGAGCTGG-3′ (reverse). the primers for human ATAD3B were: 5′-TGCCCTCATCACAGTCCAAA-3′ (forward) and 5′-GGAGGAATCCAGACCCACAG-3′ (reverse). To determine the phosphorylation status of numerous interrelated phospho-kinases in HN12 cells with or without ATAD3A KO, the Proteome Profiler Human Phospho-Kinase Array Kit (R&D Systems, Minneapolis, MN) was applied according to the manufacturer’s protocol.

### Animal studies

All animal experiments were approved by the Institutional Animal Care and Use Committee (IACUC) of Augusta University. Six-week-old NOD.Cg-*Prkdcscid Il2rgtm1Wjl/SzJ* (NSG) mice were purchased from the Jackson Laboratory (Bar Harbor, ME). An orthotopic xenograft tongue tumor model was generated as we previously described [16]. Briefly, 1 × 10^5^ luciferase containing HN12 cells carrying different ATAD3A gene modifications were suspended in 50 μl of PBS/Matrigel (3:1) and injected into the anterior ~ 1/3 tongue of NSG mice under anesthesia. Twenty-one days after cell implantation, mice were imaged for bioluminescent luciferase signal using a Xenogen IVIS-200 In Vivo Imaging System (PerkinElmer, Waltham, MA). For drug treatment, 7 days after receiving ATAD3A KO or parental HN12 cells, tumor-bearing NSG mice were treated with salirasib intraperitoneally once every other day for two consecutive weeks at 30 mg/kg body weight. The mice treated with an equal volume of vehicle (PBS) were used as control. When the experiment was terminated, the primary xenografts were excised and processed for standard histological analysis with H&E staining and IHC with antibodies against p-ERK1/2 and Ki67 as we previously described [29, 30].

### TCGA data retrieval and statistics

The mRNA expression of ATAD3A from the TCGA Pan-Cancer cohort, as well as the mRNA expression from ATAD family members from the TCGA head and neck cancer cohort, were obtained using the UCSC Xena Browser (http://xena.ucsc.edu/). The data were analyzed using two-tailed Student’s *t*-test by comparing with the control group. For multiple comparisons, data were analyzed via analysis of variance (ANOVA) with the Tukey-Kramer Multiple Comparisons Test. The data obtained from three independent repetitions were presented in the form of an average and a standard deviation, and a *p*-value of < 0.05 was set as the criterion for statistical significance.

## Results

### High expression levels of ATAD3A are associated with human HNSCC development

**Fig. 1:**
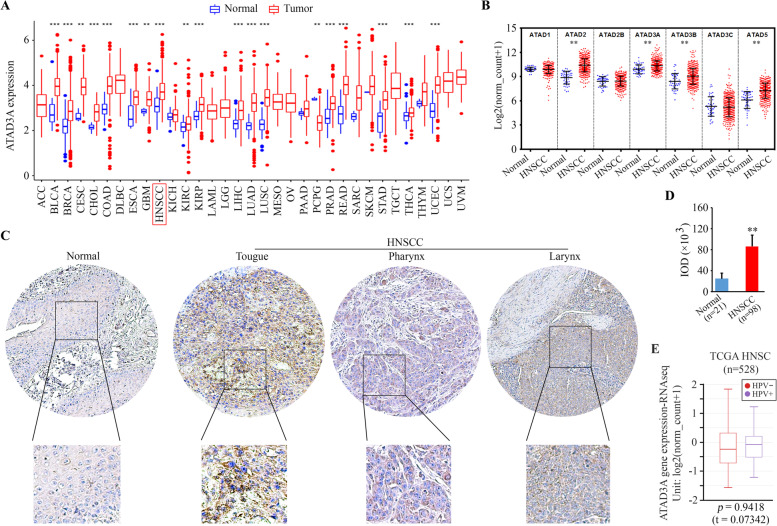
ATAD3A is highly expressed in HNSCC tissues. **A** The mRNA expression of ATAD3A within TCGA Pan-Cancer cohort (among 33 cancer types). ATAD3A shows significantly higher expression in HNSCC tissues compared with solid tissue normal samples. **B** Distribution of the expression of ATAD family members within TCGA HNSCC cases. **C**, **D** The protein levels of ATAD3A in HNSCC tissue arrays analyzed by IHC. Representative IHC images (**C**) and quantitative data of staining intensity presented as positive area score (**D**). **E** ATAD3A expression levels in HPV^−^ and HPV^+^ HNSCC tumors illustrated by TCGA head and neck cancer cohort (*n* = 528). **p < 0.01

Investigation of ATAD3A expression levels from TCGA Pan-Cancer analysis revealed that ATAD3A was highly expressed in many types of cancer, including HNSCC (Fig. 1A), which was confirmed by data from the Oncomine database (Supplementary Fig. S1A). We then examined the distribution of ATAD3A expression levels within TCGA HNSCC cases. This analysis showed ATAD3A was one of four ATAD family members that were upregulated in HNSCC (Fig. 1B). Moreover, the expression levels of ATAD3A were higher in advanced tumor stages (II, III, and IV) than in Stage I of HNSCC, although there was no statistical significance (Supplementary Fig. S1B). To verify whether ATAD3A protein expression corresponded with the observations of high mRNA expression changes, we compared the levels of ATAD3A protein in HNSCC and normal tissues using tissue microarrays. Significantly higher levels of ATAD3A were observed in primary HNSCC tissues, including the tongue, pharynx, and larynx, as illustrated by IHC with anti-ATAD3A antibody (Fig. 1C and D). We also examined the expression levels of ATAD3A in human papillomavirus (HPV) negative (−) and positive (+) HNSCC using the gene expression data from TCGA head and neck cancer cohort (Fig. 1E). There was no statistically significant difference in ATAD3A expression levels between HPV^−^ and HPV^+^ tumor groups, suggesting that it is not specifically associated with HPV infection status. These findings support the involvement of ATAD3A in HNSCC development and progression.

### Loss of ATAD3A gene expression impairs mitochondrial functions and subsequently constrains HNSCC development

**Fig. 2:**
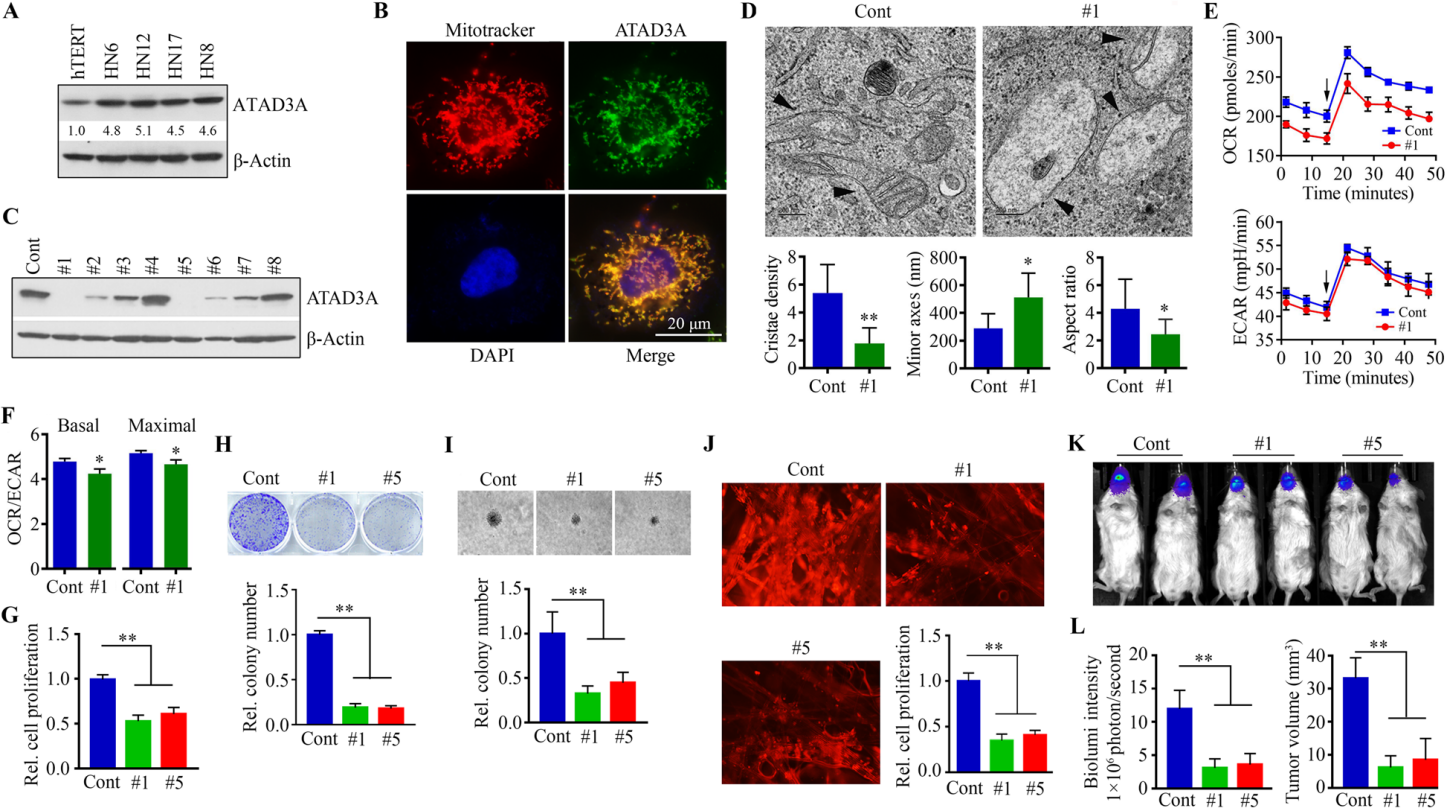
Loss of ATAD3A impairs mitochondrial function in HNSCC cells and inhibits tumor development. **A** ATAD3A protein levels in various HNSCC cell lines and normal oral keratinocytes (hTERT). **B** Subcellular localization of ATAD3A in HN12 cells determined by confocal microscopy after IF staining. **C** ATAD3A KO clones derived from the sgATAD3A-targeting HN12 cells screened by Western blotting. **D** Mitochondrial structure of ATAD3A KO (#1) and parental HN12 cells assessed by TEM. Mitochondrial cristae density, minor axes and aspect ratio (major axis/minor axis) were quantified from 20 randomly selected fields. Arrow heads indicate mitochondria. **E** OCR and ECAR profiles of ATAD3A KO (#1) and parental HN12 cells measured by a Seahorse XFe96 analyzer. In this assay, 1 μM Oligo that inhibits ATP synthesis and 1 μM FCCP that uncouples OXPHOS were injected at the indicated point (arrow). **F** OCR:ECAR ratio at basal and maximal respiration in ATAD3A KO and parental HN12 cells. **G** The effect of ATAD3A KO (#1 and #5) on cell proliferation on Day 3. **H** The effect of ATAD3A KO on cell colony formation within 3 weeks. Quantitative data from colony formation are shown in the lower panel (*n* = 3). **I** The effect of ATAD3A KO on soft agar cell growth on Day 14. Quantitative data from soft agar assays are shown in the lower panel (*n* = 3). **J** The effect of ATAD3A KO on HN12 cell growth on Day 14 in 3D SeedEZ scaffold. Quantitative data from 3D assays are shown in the right lower panel (*n* = 3). **K** Representative bioluminescence images showing the effect of ATAD3A KO on HN12-derived tongue tumor growth in NSG mice. Tumor progression was monitored on Day 15 by examining bioluminescence in Xenogen IVIS-200 In Vivo Imaging System. **L** Quantitative data of bioluminescence intensity and tumor volume in the indicated groups (*n* = 5 mice/group). hTERT: Human telomerase-immortalized tonsillar keratinocytes hTERT HAK Clone 41. *p < 0.05; **p < 0.01

Next, we determined the levels of ATAD3A protein in various HNSCC cells and found that ATAD3A was expressed in all cell lines examined (Fig. 2A). Interestingly, the levels of ATAD3A were stronger in all HNSCC cell lines compared with oral keratinocytes (Fig. 2A). IF assay showed that ATAD3A localized to mitochondria in HNSCC cells as evidenced by co-localization between the MitoTracker signal and the signal produced from anti-ATAD3A antibody (Fig. 2B). To better understand the role of ATAD3A in HNSCC, we generated ATAD3A KO HN12 cells using the CRISPR/Cas9 gene-editing system (Supplementary Fig. S2). Clone screening of ATAD3A KO by Western blotting showed that clones #1 and #5 from HN12 cells experienced a complete loss of ATAD3A expression (Fig. 2C) and, therefore, they were used for subsequent study.

We first examined the alterations in mitochondrial functions in HNSCC cells with or without ATAD3A KO. TEM revealed that loss of ATAD3A in HN12 cells led to significant changes in mitochondrial structure, showing a high reduction in the number of normally shaped mitochondria with decreased mitochondrial cristae density (Fig. 2D). It was also apparent that depletion of ATAD3A decreased mitochondrial aspect ratio (major axes/minor axes), reflecting so-called fragmentation (Fig. 2D). Since inhibition of mitochondrial fusion may result in oxidative phosphorylation (OXPHOS) impairment, we then assessed the impact of ATAD3A loss on mitochondrial metabolic function by measuring OCR and ECAR, two indicators of cellular respiration and glycolysis respectively. A sustained decrease in baseline OCR level was observed in ATAD3A KO HN12 cells compared with parental cells, which remained relatively lower level even following exposure to Oligo and FCCP (Fig. 2E). Both basal and stimulated ECAR levels were slightly lower in ATAD3A KO HN12 cells than in parental cells (Fig. 2E). Most importantly, a significant decrease in OCR:ECAR ratio at basal and maximal respiration was seen in HN12 cells when ATAD3A was depleted (Fig. 2F), suggesting that ATAD3A-deficient HNSCC cells are more reliant on glycolysis for growth.

Consistent with the functional changes of mitochondria, ATAD3A KO (#1 and #5) remarkably reduced the potential of cell proliferation and colony formation compared with parental cells (Fig. 2G and H). DNA flow cytometry was performed to ascertain the cell cycle distribution of growing HN12 cells after ATAD3A depletion. The distribution profile showed that loss of ATAD3A induced S phase cell cycle arrest, as evidenced by a slight but significant accumulation of cell population in this phase (Supplementary Fig. S3A). There were no noticeable changes in the protein levels of cyclin D1 and CDK4 between ATAD3A KO and parental HN12 cells (Supplementary Fig. S3C), suggesting that ATAD3A suppresses cell growth by mechanisms other than directly affecting the major cell cycle checkpoint factors. Additionally, ATAD3A KO could not trigger apoptosis in HN12 cells as cleaved caspase 3 was not detected in these cells (Supplementary Fig. S3C). Soft agar colony formation assays further revealed that either the colony size or number was significantly reduced in ATAD3A KO cells (Fig. 2I), indicating that loss of ATAD3A abrogates the ability for HNSCC cell anchorage-independent growth. It is well accepted that 3D cell cultures hold promise to avoid certain drawbacks of 2D cultures, by allowing cells to replicate in vitro while providing an accurate representation of cell growth in vivo [31, 32]. Next, we determined the effect of ATAD3A KO on the 3D growth of HN12 cells using SeedEZ scaffold and found that inhibition of ATAD3A significantly suppressed cell growth when compared with the parental cells (Fig. 2J). To determine whether this was also the case in other HNSCC cells, HN8 and HN17 cells were used to generate stable ATAD3A knockdown cells. Lentivirus-mediated shRNAs remarkably decreased ATAD3A expression levels and led to a reduction in both cell proliferation and the capacity for colony formation (Supplementary Fig. S4), suggesting that ATAD3A promotes cell growth in a broad range of HNSCC cells. Our previous study has demonstrated that loss of ATAD3A suppresses invasion potential in breast cancer cells [3]. Similar results were observed in ATAD3A KO HN12 cells, and reduced cell invasion was accompanied by increased E-cadherin protein levels and decreased vimentin protein levels compared with parental cells (Supplementary Fig. S3B and C).

To understand the in vivo role of ATAD3A in HNSCC, we generated an orthotopic mouse model of HNSCC by injecting 1 × 10^5^ luciferase containing HN12 cells (HN12-luc) into the anterior ~ 1/3 tongue of NSG mice. Three weeks after inoculation, the tumor size in the mice receiving ATAD3A KO cells was significantly smaller compared with that in mice receiving the parental cells, as measured by bioluminescence and tumor volume (Fig. 2K and L). These results indicate that inhibition of ATAD3A can largely constrain HNSCC.

### ATAD3A WA dead mutant impairs its oncogenic function in HNSCC cells

**Fig. 3:**
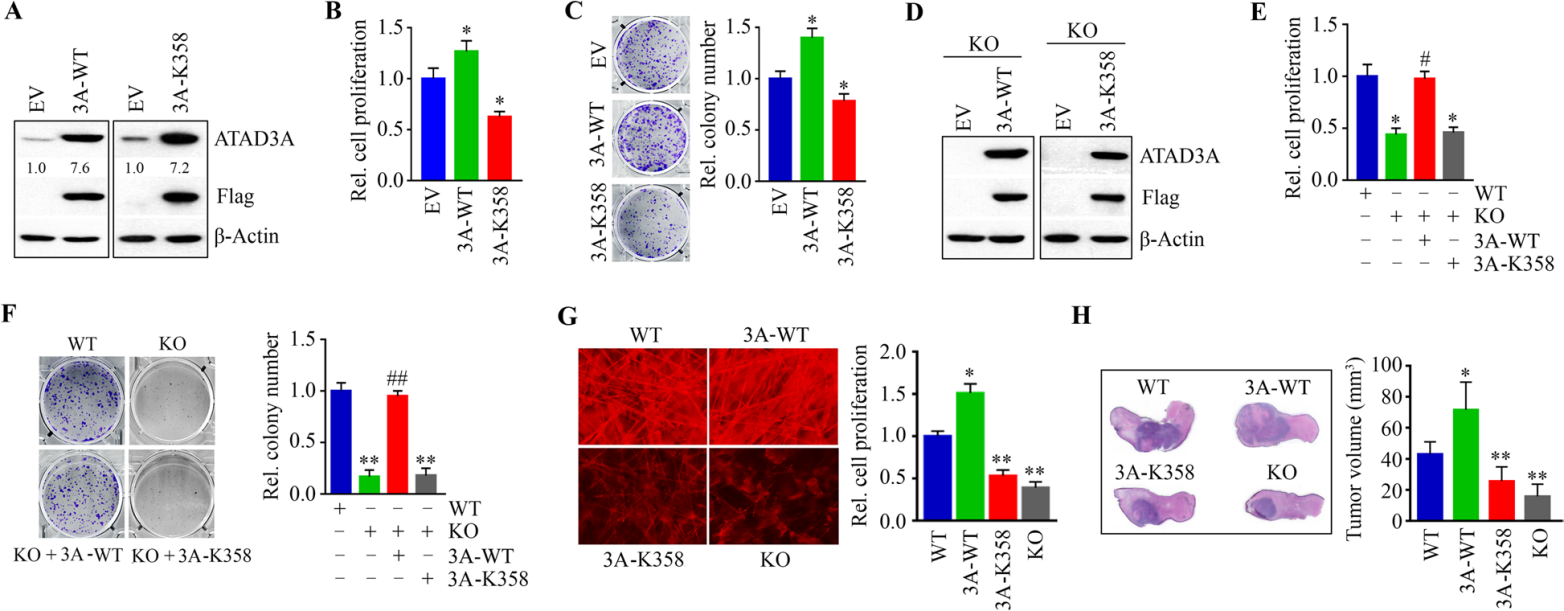
ATAD3A WA dead mutant functions as a dominant-negative for its oncogenic role in HNSCC cells. **A** Overexpression of ATAD3A and its WA dead mutant (K358A) in HN12 cells determined by Western blotting. **B** The effect of overexpression of ATAD3A and its WA dead mutant on cell proliferation on Day 3 in HN12 cells. **C** The effect of overexpression of ATAD3A and its WA dead mutant on cell colony formation within 3 weeks in HN12 cells. Quantitative data from colony formation assays are shown in the right panel (*n* = 3). **D** Overexpression of ATAD3A and its WA dead mutant in ATAD3A KO HN12 cells determined by Western blotting. **E** The effect of overexpression of ATAD3A and its WA dead mutant on cell proliferation on Day 3 in ATAD3A KO HN12 cells. **F** The effect of overexpression of ATAD3A and its WA dead mutant on cell colony formation within 3 weeks in ATAD3A KO HN12 cells. Quantitative data from colony formation assays are shown in the right panel (*n* = 3). **G** The effects of ATAD3A KO and overexpression of ATAD3A or its WA dead mutant on 3D cell growth on Day 14 in HN12 cells. Quantitative data from alamarBlue staining are shown in the right panel (*n* = 3). **H** The effect of ATAD3A WA dead mutant on HN12-derived tongue tumor growth in NSG mice. Representative scanning images of mice tongue with H&E staining and quantitative tumor volume (*n* = 5 mice/group) are shown in the right and left panels, respectively. WT: wild-type cells; KO: ATAD3A knockout cells; EV: cells overexpressing empty vector; 3A-WT: cells overexpressing wild-type ATAD3A; 3A-K358: cells overexpressing ATAD3A WA dead mutant. *p < 0.05 versus EV or WT; **p < 0.01 versus EV or WT; #*p* < 0.05 versus KO; ##*p* < 0.01 versus KO

In contrast, overexpression of ATAD3A counteracted the effects observed in ATAD3A depleted cells, as evidenced by increased cell proliferation and enhanced cell colony formation (Fig. 3A-C). To clarify the importance of the WA motif in ATAD3A-mediated oncogenic function, we generated a WA dead mutant of ATAD3A (K358) lacking ATP binding ability. Overexpression of this mutant significantly decreased proliferation and colony number compared with control cells, contrasting the effects observed in ATAD3A-overexpressing cells (Fig. 3A-C). To minimize off-target effects, ATAD3A was overexpressed in ATAD3A KO HN12 cells (#1 pooled with #5). While the rescue of ATAD3A expression in ATAD3A KO cells restored cell proliferation and colony-forming ability (Fig. 3D-F), no changes were detected in cell proliferation and colony formation in ATAD3A KO cells overexpressing ATAD3A WA dead mutant (Fig. 3D-F). Consistent data was also seen in cells growing in SeedEZ scaffolds (Fig. 3G). These observations indicate that the ATP-binding ability of ATAD3A is required for its oncogenic role in HNSCC cells.

Next, we generated xenograft tongue tumors in NSG mice through injection of luciferase containing HNSCC cells into the anterior tongue of mice [16]. A marked increase in tumor growth was observed in mice that were implanted with ATAD3A-overexpressing HN12 cells compared with mice receiving parental cells (Fig. 3H). In line with in vitro data, either KO of ATAD3A or overexpression of its WA dead mutant significantly suppressed tumor outgrowth in mice, as evidenced by reduced bioluminescence intensity and tumor size compared with the other two groups (Fig. 3H). Collectively, these novel data demonstrate that ATAD3A controls HNSCC growth in a manner dependent on its ATP-binding ability.

### ATAD3A regulates mitochondrial ERK1/2 signaling in HNSCC cells

**Fig. 4:**
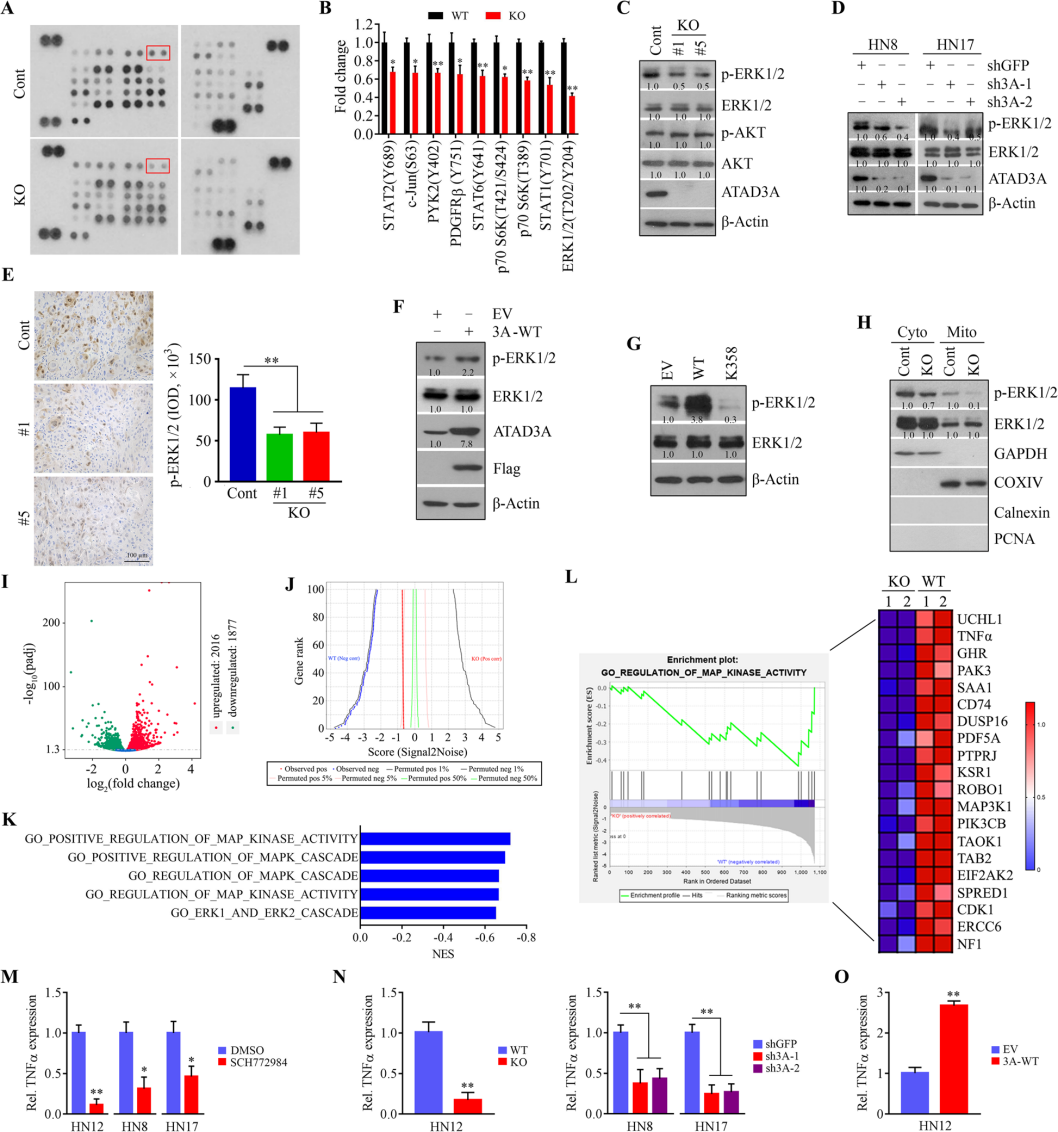
ATAD3A regulates mitochondrial ERK1/2 activation in HNSCC cells. **A**, **B** The effect of ATAD3A KO on the activation of phospho-kinases in HN12 cells determined by Human Phospho-Kinase array. Representative images and quantitative data (*n* = 2) are respectively shown in (**A**) and (**B**). **C** The effect of ATAD3A KO on ERK1/2 inactivation validated in HN12 cells by Western blotting. **D** The effect of ATAD3A KO on ERK1/2 inactivation in HN8 and HN17 cells. **E** The phosphorylation levels of ERK1/2 in tumor xenografts from mice implanted with ATAD3A KO or parental HN12 cells determined by IHC. Representative images and quantitative data are shown in the left and right panels, respectively. **F** The effect of ATAD3A overexpression on ERK1/2 activation. **G** The effect of ATAD3A WA dead mutant on ERK1/2 activation. **H** The effect of ATAD3A KO on the cytosolic and mitochondrial ERK1/2 activation in HN12 cells. The purity of the mitochondrial fractions was indicated by the mitochondrial COXIV protein and the absence of cytosolic GAPDH protein, nuclear PCNA protein and ER-localized Calnexin protein. **I**, **J** Volcano (**I**) and butterfly (**J**) plots resulted from the differential expression analysis between ATAD3A KO and parental HN12 cells. **K** GO enrichment analysis of DEGs resulting from ATAD3A loss showing downregulation of the ERK1/2 related pathways upon ATAD3A KO in HN12 cells. **L** A down-regulated enrichment plot for a priori gene sets for the regulation of MAPK activity. **M** The effect of SCH772984 on TNFα expression in three HNSCC cell lines determined by RT-qPCR. **N** The effect of ATAD3A loss on TNFα expression in three HNSCC cell lines determined by RT-qPCR. **O** The effect of ATAD3A overexpression on TNFα expression in three HNSCC cell lines determined by RT-qPCR. Cyto: cytosol; Mito: mitochondria. *p < 0.05; **p < 0.01

Next, we performed Proteome Profiler Human Phospho-Kinase Array assays for the parallel assessment of the relative phosphorylation levels of 43 human protein kinases upon ATAD3A loss. Of these, the phosphorylation of nine protein kinases was significantly downregulated in HN12 cells when ATAD3A was knocked out (Fig. 4A and B). Among them, ERK1/2 was the protein kinase most inactivated in ATAD3A knockdown HN12 cells (Fig. 4A and B), which was confirmed by Western blotting (Fig. 4C). Dephosphorylation of ERK1/2 resulting from ATAD3A loss did not affect the phosphor-AKT levels in HN12 cells (Fig. 4C). Additionally, decreased phospho-ERK1/2 levels were seen in HN6 and HN17 cells when ATAD3A was depleted (Fig. 4D), suggesting this is not a cell-type-specific effect. IHC data from mice tongue tumors derived from ATAD3A KO and parental HN12 cells validated that the loss of ATAD3A significantly inhibited ERK1/2 phosphorylation in xenograft tumors (Fig. 4E). In contrast, ATAD3A overexpression increased phospho-ERK1/2 levels in HN12 cells (Fig. 4F), suggesting that ATAD3A has the potential to activate ERK1/2 in HNSCC cells. We then focused on studying the regulation of ERK1/2 by ATAD3A. Interestingly, ATAD3A WA dead mutant decreased ERK1/2 phosphorylation (Fig. 4G), which was in line with the observations of deceased cell growth (Fig. 3B and C). Western blotting data further revealed the presence of ERK1/2 in the mitochondria of HN12 cells, and ATAD3A knockdown was seen to almost completely repress ERK1/2 phosphoactivation in the mitochondria (Fig. 4H), suggesting that ATAD3A primarily regulates mitochondrial ERK1/2 activation.

To explore changes in gene expression occurring in cells with or without ATAD3A loss, we carried out whole-transcriptome sequencing studies, which showed that 1475 genes were upregulated and 1401 genes were downregulated in ATAD3A KO HN12 cells (Fig. 4I and J). GO pathway analysis revealed differential enrichment for multiple functions upon comparison of genes downregulated in ATAD3A KO cells with controls (Fig. 4K). Intriguingly, downregulation of the ERK1/2 related pathways was seen in ATAD3A KO HN12 cells (Fig. 4K and L). As shown in Fig. 4L, TNFα, a well-known ERK1/2 downstream target, was identified as one of the top three downregulated genes involved in the MAPK pathway upon ATAD3A loss. RT-qPCR confirmed that TNFα was downregulated both in cells treated with ERK1/2 inhibitor SCH772984 (Fig. 4M) and in cells with ATAD3A depletion (Fig. 4N). As expected, elevated TNFα expression levels were seen in ATAD3A-overexpressing HN12 cells (Fig. 4O). Collectively, these observations indicate that ATAD3A regulates MAPK activity through modulating mitochondrial ERK1/2 activation in HNSCC cells.

### ATAD3A interacts with the mitochondrial ERK1/2 via VDAC1 in HNSCC cells

**Fig. 5:**
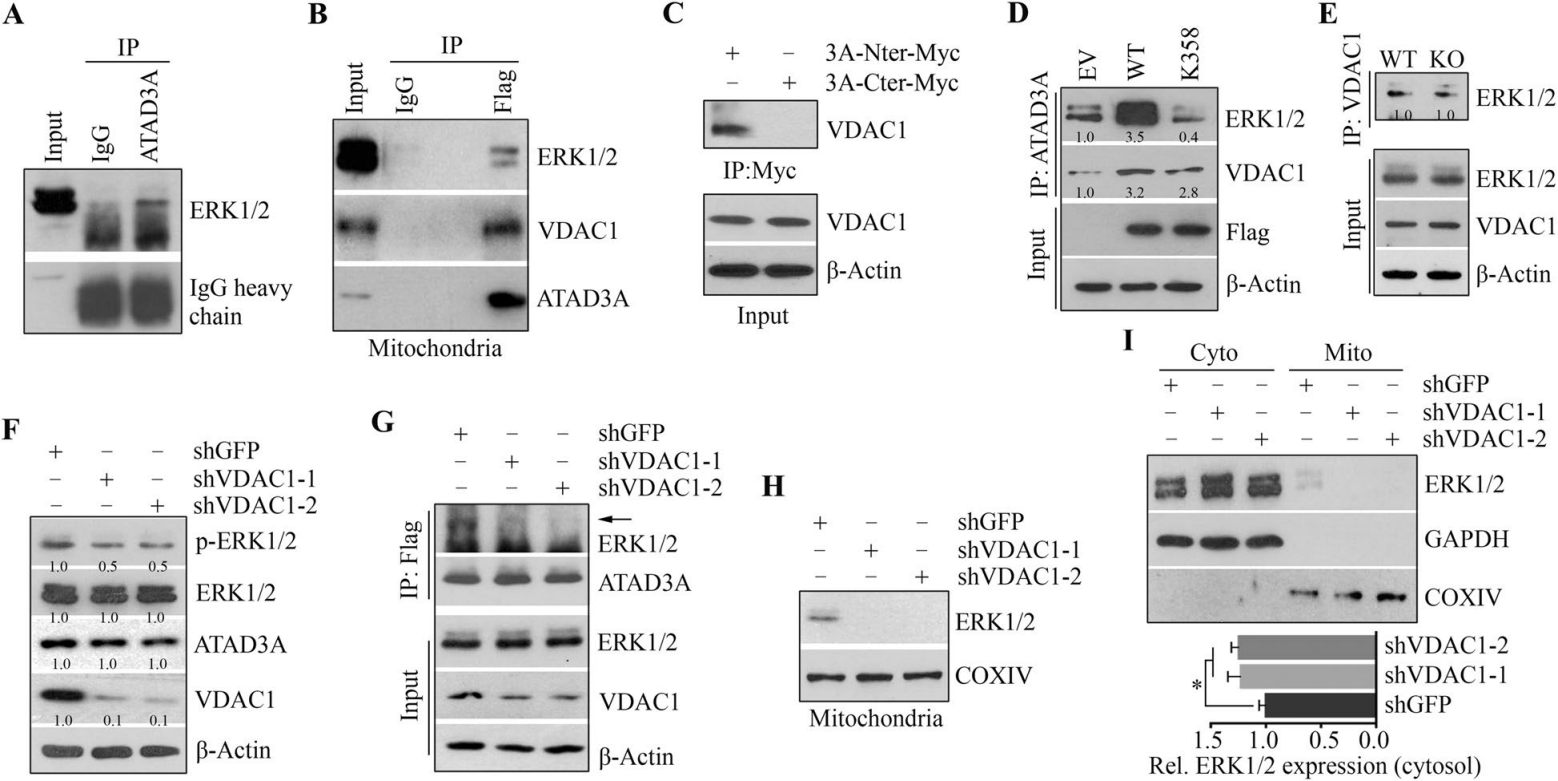
ATAD3A interacts with mitochondrial ERK1/2 in a VDAC1-dependent manner. **A** The interaction between ATAD3A and ERK12 proteins in HN12 cells determined by IP. **B** The presence of VDAC1 in the ATAD3A-ERK1/2 immunocomplex in HN12 cell mitochondria. **C** The binding of ATAD3A N-terminus (Nter, 1-287 aa) and C-terminus (Cter, 258-586 aa) to VDAC1 protein in HN12 cells. **D** The necessity of the ATPase activity of ATAD3A for ATAD3A-ERK1/2 interaction in HN12 cells. **E** The effect of ATAD3A KO on the interaction between VDAC1 and ERK1/2. **F** The effect of VDAC1 knockdown on ERK1/2 activation in HN12 cells. **G** The effect of VDAC1 knockdown on the interaction between ATAD3A and ERK1/2 proteins in ATAD3A-overexpressing HN12 cells. The immunoprecipitates were pulled down using an anti-Flag antibody. **H** The effect of VDAC1 knockdown on the levels of mitochondrial ERK1/2 in HN12 cells. **I** The effect of VDAC1 on the cytosolic and mitochondrial ERK1/2 protein levels in HN12 cells. The purity of the mitochondrial fractions was indicated by the mitochondrial COXIV protein and the absence of the cytosolic GAPDH protein. Representative results and quantitative data from three independent experiments are shown in the upper and lower panels, respectively. *p < 0.05; **p < 0.01

ATAD3A may regulate ERK1/2 activation by protein-protein interactions. To test this possibility, an anti-ATAD3A antibody was used to IP the ATAD3A protein complex from HN12 cells, followed by Western blotting with an anti-ERK1/2 antibody. This assay identified the presence of ERK1/2 in the ATAD3A immunocomplex (Fig. 5A). To determine whether the interaction between ATAD3A and ERK1/2 is maintained in the mitochondria, mitochondria fractions were isolated from ATAD3A-overexpressing HN12 cells and the exogenous ATAD3A protein was immunoprecipitated by the Flag antibody. Intriguingly, the ERK1/2-ATAD3A interaction was found in the mitochondria (Fig. 5B), suggesting that ATAD3A binds to the mitochondrial ERK1/2 in HNSCC cells. Higher levels of ERK1/2 protein were seen in the ATAD3A immunocomplex from HN12 cells overexpressing wild-type ATAD3A compared with cells overexpressing ATAD3A K358 mutant (Fig. 5D), suggesting that binding of ATAD3A to ATP is a necessary step to facilitate ERK-ATAD3A interaction and the mutation of ATAD3A at K358 represses ERK1/2 activation through impairing its interaction with ERK1/2.

VDAC1 is one protein known to interact with ATAD3A [5, 33], which is the main channel of the MOM [17, 34]. IP confirmed that VDAC1 was present in the ATAD3A immunocomplexes in HN12 cells (Fig. 5B). It is worth mentioning that ATAD3A has two transmembrane regions: transmembrane domain 1 (TM1, 225-242 aa) for integrating with MOM and transmembrane domain 2 (TM2, 247-264 aa) for integrating with MIM [5]. Further analysis showed that ATAD3A only interacted with VDAC1 at its N-terminal part (1-287 aa) (Fig. 5C), supporting the notion that these two proteins are bound together on the MOM in HNSCC cells. The binding of ATAD3A to VDAC1 was independent of its ATPase activity, as no decreased amount of VDAC1 was present in the ATAD3A immunocomplex regardless of the overexpression of ATAD3A wild-type or K358 mutant (Fig. 5D). In addition, loss of ATAD3A in HN12 cells did not affect the binding of VDAC1 to ERK1/2 (Fig. 5E). To determine whether VDAC1 is required for the interaction between ATAD3A and ERK1/2, we depleted it by shRNAs. Knockdown of VDAC1 in HN12 cells significantly decreased cell proliferation and impaired the capability of a single cell to grow into a large colony (Supplementary Fig. S5), which was also associated with reduced ERK1/2 phosphorylation levels (Fig. 5F). Loss of VDAC1 did not affect the total ATAD3A and ERK1/2 protein levels (Fig. 5F); however, it disrupted the ERK1/2-ATAD3A interaction, as evidenced by a remarkable reduction in the binding of ERK1/2 to ATAD3A (Fig. 5G and Supplementary Fig. S6). Moreover, there was no ERK1/2 detected in the mitochondria of HN12 cells where VDAC1 was depleted (Fig. 5H). There was a possibility that VDAC1 might facilitate the mitochondrial transport of ERK1/2. To test this, the mitochondrial and cytosolic fractions were isolated from VDAC1 knockdown and control HN12 cells and then subjected to Western blotting analysis. Strikingly, a statistically significant increase in cytosolic ERK1/2 levels was observed in VDAC1-depleted cells when compared with the knockdown control cells, which was associated with the absence of mitochondrial ERK1/2 (Fig. 5I). Taken together, these findings suggest that VDAC1 promotes ERK1/2 mitochondrial transport which is essential for the formation of ATAD3A-ERK1/2 protein complex in the HNSCC cell mitochondria.

### ATAD3A regulates mitochondrial ERK1/2 activation in a RAS-independent fashion

**Fig. 6:**
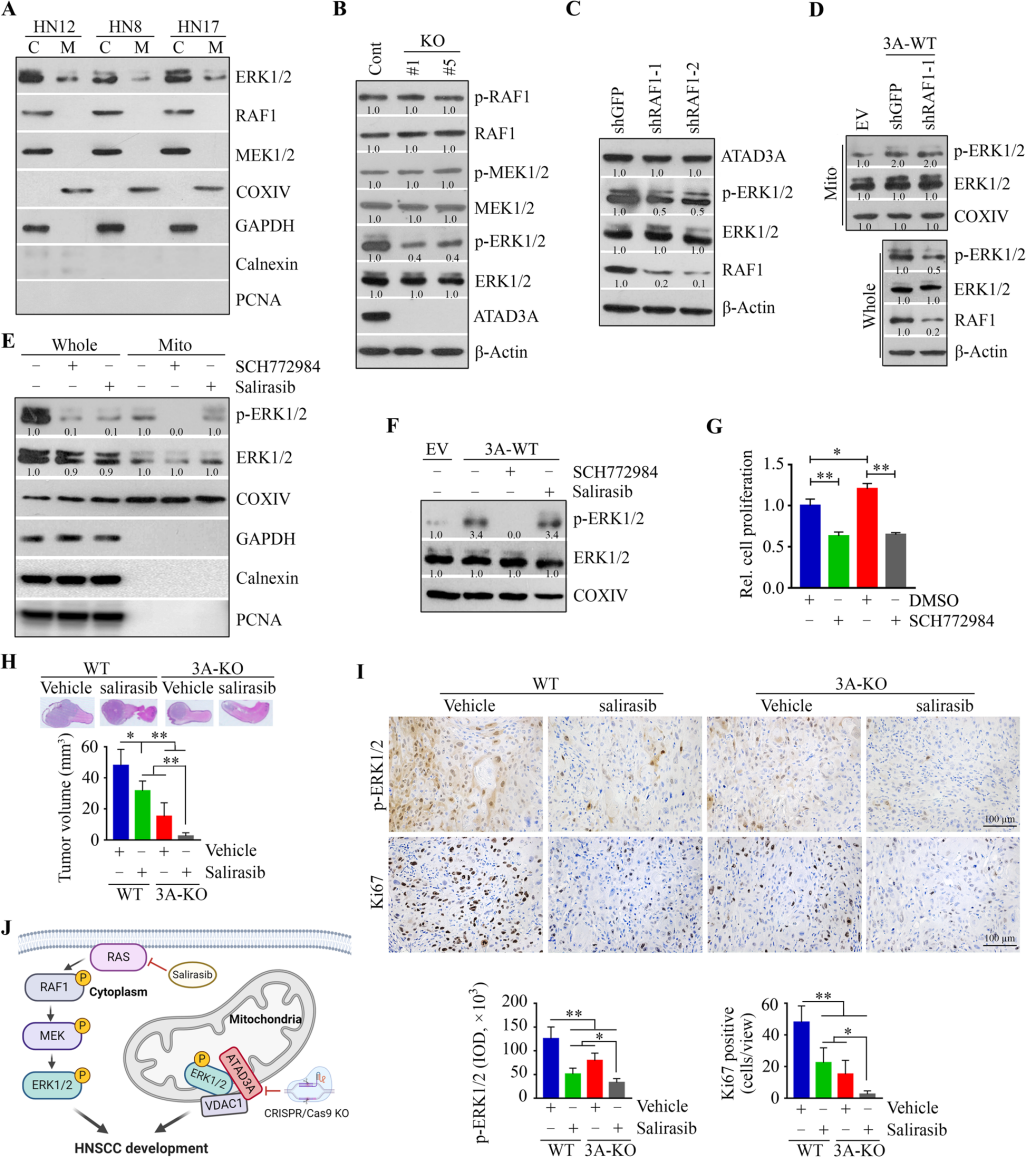
ATAD3A regulates mitochondrial ERK1/2 activation via RAS-independent mechanisms and co-inhibiting ATAD3A and RAS signaling pathways exhibits a synergistic anti-HNSCC effect. **A** The presence of RAF1, MEK1/2, and ERK1/2 in the mitochondria of three HNSCC cell lines. **B** The effect of ATAD3A KO on RAF1 and MEK activation in HN12 cells. **C** The effect of RAF1 knockdown on ATAD3A and ERK1/2 phosphorylation in HN12 cells. **D** The effect of RAF1 knockdown on mitochondrial ERK1/2 phosphorylation in ATAD3A-overexpressing HN12 cells. **E** The effect of RAS inhibitor salirasib (50 μM) and ERK1/2 inhibitor SCH772984 (1 μM) on ERK1/2 phosphorylation in HN12 cells. **F** The effect of salirasib and SCH772984 on mitochondrial ERK1/2 phosphorylation in ATAD3A-overexpressing HN12 cells. **G** The effect of SCH772984 on cell proliferation in ATAD3A overexpressing or parental HN12 cells. **H** The synergistic effect of salirasib treatment and ATAD3A KO on HN12-derived tongue tumor growth in NSG mice. Tumor progression was monitored on day 14 after treatment initiation by examining bioluminescence in Xenogen IVIS-200 In Vivo Imaging System. Representative bioluminescence images and quantitative data (*n* = 5 mice/group) are respectively shown in upper and lower panels. **I** The phosphorylation levels of ERK1/2 and Ki67-positive cells in xenograft tumors derived from mice receiving the indicated treatment were determined by IHC. Representative images and quantitative data are shown in the upper and lower panels, respectively. **J** Proposed model for the synergistic effect of cytoplasmic RAS/RAF1/MEK/ERK signaling and mitochondrial ATAD3A-ERK1/2 signaling in HNSCC development. Whole: whole cells; C: cytosol; M or Mito: mitochondria. *p < 0.05; **p < 0.01

The ERK cascade controlled by RAS-RAF-MEK signaling has been well studied [35]. There was a possibility that the mitochondrial ERK1/2 regulated by ATAD3A was under the control of RAS-RAF-MEK signaling. To address this, we first examined the subcellular localization of RAF1 and MEK1/2 by treating isolated mitochondria of HN12 cells with the exogenous protease trypsin. The presence of mitochondrial COXIV and the absence of the common cytoplasmic GAPDH demonstrated the purity of the mitochondrial fraction (Fig. 6A). Intriguingly, ERK1/2, but not RAF1 and MEK1/2, was present in HN12 cell mitochondria (Fig. 6A), suggesting that ATAD3A-mediated ERK1/2 activation may be independent of the RAS-RAF-MEK pathway. Moreover, KO of ATAD3A did not affect the phosphorylation levels of RAF1 and MEK1/2 (Fig. 6B). Depletion of RAF1 suppressed total ERK1/2 phosphorylation without affecting ATAD3A protein levels in HN12 cells, regardless of whether ATAD3A was overexpressed or not (Fig. 6C and D). However, RAF1 knockdown could not attenuate the mitochondrial ERK1/2 phosphorylation that was induced by ATAD3A overexpression (Fig. 6C and D). In line with these observations, both the ERK1/2 inhibitor SCH772984 and RAS inhibitor salirasib induced the repression of total ERK1/2 phosphorylation in HN12 cells, but only SCH772984 inhibited mitochondrial ERK1/2 phosphorylation (Fig. 6E). While SCH772984 dramatically abrogated ATAD3A-mediated increase of mitochondrial ERK1/2 phosphorylation (Fig. 6F) and attenuated the proliferation induced by ATAD3A overexpression (Fig. 6G), there were no changes in the phosphorylation levels of mitochondrial ERK1/2 when ATAD3A-overexpressing HN12 cells were treated with salirasib (Fig. 6F). Collectively, these findings demonstrate that ATAD3A promotes HNSCC development at least partially via RAS-independent ERK1/2 activation.

### Co-inhibition of ATAD3A and RAS signaling pathways achieves greater anti-HNSCC efficacy than singly suppressing either pathway

Next, we generated orthotopic tongue tumors in mice to evaluate the synergistic effect of salirasib and ATAD3A genetic depletion on tumor growth. After a two-week treatment, tumor burden was reduced in mice treated with salirasib compared with vehicle treatment (Fig. 6H). Intriguingly, salirasib-induced repression in tumor growth was more significant in mice receiving ATAD3A KO HN12 cells than those implanted with the parental cells (Fig. 6H), indicating that salirasib achieves a better outcome in oral tumors with loss of ATAD3A expression. To determine whether ERK1/2 signaling contributes to this synergistic effect, we measured ERK1/2 activation in xenograft tumors derived from the aforementioned treatments. IHC analysis demonstrated that the depletion of ATAD3A augmented the decrease in ERK1/2 phosphorylation levels in salirasib treated tumors (Fig. 6I). Moreover, salirasib was effective in inhibiting tumor cell proliferation in mice and synergized with ATAD3A loss in hampering tumor growth, as illustrated by the lowest number of Ki67-positive cells in xenograft tumors derived from mice receiving salirasib treatment and ATAD3A gene depletion (Fig. 6I). These findings indicate that co-inactivating cytosolic and mitochondrial forms of ERK1/2 may have a superior effect on oral tumor regression (Fig. 6J).

## Discussion

HNSCC remains a lethal disease despite concerted efforts to improve its diagnosis and treatment [36]. Although there are no or few ATAD3A mutants reported in human cancers, the overexpression of ATAD3A has been observed in a high percentage of primary HNSCC tumors, including those HNSCC patients with the worst prognosis. Thus, there is a great need to understand the molecular mechanisms by which ATAD3A drives HNSCC malignancy. In the present study, we show that mitochondrial ATAD3A acts as an oncogene to promote HNSCC cell growth and inhibiting it by genetic depletion or overexpression of the WA dead mutant induces head and neck tumor regression. Most importantly, we identify a novel mechanism that ATAD3A exhibits its oncogenic role through activating mitochondrial ERK1/2 in a RAS-independent fashion.

HPV infection and tobacco smoking are well-known risk factors for HNSCC [37]. Our TCGA analysis has shown no significant difference in ATAD3A expression levels between HPV^−^ and HPV^+^ HNSCC subtypes. To determine the ATAD3A expression status under the influence of tobacco, we treated HNSCC cell lines HN12 and HN8 with a smoking mimetic 4NQO at a dose of 100 μg/ml for 24 h. Interestingly, 4NQO augments ATAD3A expression in both cell lines (Supplementary Fig. S7), suggesting that tobacco may function as an environmental trigger to upregulate ATAD3A expression in HNSCC cells. With this information, one of our future research focuses is to understand the clinical significance of ATAD3A in HNSCC patients with tobacco use and explore mechanisms underlying its contribution to smoking-related cancer development.

ATAD3A has two gene family members: ATAD3B and ATAD3C. Our TCGA analysis also shows elevated ATAD3B expression levels in HNSCC tissues relative to normal counterparts, which is supported by our experimental results from HNSCC cell lines and normal oral keratinocytes (Supplementary Fig. S8A). ATAD3A is ubiquitously expressed in all tissues from very early embryonic stages to adulthood, while ATAD3B is specifically expressed in human embryonic stem cells and is re-expressed in certain types of cancers [1, 5, 38, 39]. It appears that ATAD3B gains more expression in HNSCC cells. Merle et al. has discovered that ATAD3B functions as dominant negative for the ubiquitous ATAD3A [40]. Recently, Shu and his colleagues reported that ATAD3B hetero-oligomerizes with ATAD3A in mitochondria, which may cooperate with or antagonize ATAD3A functions [38]. Nevertheless, no evidence shows the regulation of ATAD3B by ATAD3A. In the present study, we did not observe the reduction of ATAD3B expression levels in ATAD3A KO HN12 cells (Supplementary Fig. S8B), excluding the possibility that ATAD3A affects ATAD3B function at the mRNA level.

Mitochondria constitute promising targets for the development of novel anticancer agents as they are a point of integration for signaling cascades which impact virtually all processes linked to oncogenesis, encompassing malignant transformation, tumor progression, and metastasis [1, 41]. Of course, non-specifically targeting mitochondria may have major unwarranted effects in cancer treatment, including the inhibition of normal cell growth. Therefore, refined strategies that allow for specifically blocking the function of oncoproteins that physically localize to the mitochondria in cancer cells will have to be devised for therapeutics [2, 42]. Given that there is no simple standard for the role of mitochondrial oncoproteins in the regulation of malignant mitochondrial programs, gaining mechanistic insights into these proteins and their signaling network involved in the main stages of tumor development will be critical for the clinical exploration of novel anticancer therapies. ATAD3A is one such oncoprotein contributing to mitochondrial dynamics, nucleoid organization, protein translation, cell growth, and cholesterol metabolism [3, 12, 19, 43], which is understudied in cancers. At this stage of our understanding, ATAD3A dysfunction is required and sufficient to drive the oncogenic process of HNSCC, and depleting it has significant therapeutic effects in reducing tumor burden. These results, therefore, indicate an as yet undescribed functional relevance of ATAD3A in HNSCC and provide a molecular basis for targeting ATAD3A to control HNSCC development, which may interest a range of cancer scientists and clinicians who seek to assess the feasibility of manipulating mitochondrial signaling for therapeutic purposes.

Recurrent de novo and biallelic variation of ATAD3A have been implicated to result in distinct neurological syndromes, providing an additional link between the dysfunction of mitochondrial proteins and recognizable diseases [44]. The WA^K358A^ mutant is the WA dead mutant of ATAD3A and when it is expressed, it becomes incapable of ATP binding, leading to fragmentation of the mitochondrial network in human glioma U373 cells and mouse NIH 3 T3 cells [5, 7]. In our study, we demonstrate that the ATP-binding ability of ATAD3A strictly regulates the ATAD3A-ERK1/2 signaling axis and enables its role in favor of HNSCC growth by overexpressing WA^K358A^. We also tested the necessity of the WB motif using the WB mutant of ATAD3A (E413Q, substitution of glutamine 413 to glutamate) that binds to ATP but is defective in ATP hydrolysis [7]. Expression of WB^E413Q^ does not result in phenotypes similar to those of WA^K358A^, supporting the notion that the WA domain, but not the WB domain, is required for the oncogenic role of ATAD3A in HNSCC cells. G355D (substitution of glycine 355 to aspartate) is another WA mutant of ATAD3A representing patients with hereditary spastic paraplegia and tetraplegia [6]. This mutant changes the affinity of the substrate-binding site for ATP and has a strong dominant-negative effect on the ATPase activity of ATAD3A [6]. Whether the WA^G355D^ mutant has the same function as WA^K358A^ in HNSCC cells, however, remains to be explored. In addition, Baudier’s group found a truncated 50-amino-acid N-terminus mutant functioned as a dominant-negative for participation in interactions with the mitochondrial outer membrane in glioma cells [7]. Therefore, vigorous research efforts are warranted to further clarify which mutant of ATAD3A is more profound in blocking its oncogenic signaling in HNSCC cells.

The ERK1/2 cascade is a central signaling pathway that regulates a wide variety of stimulated cellular processes. The widespread involvement of ERK1/2 dysregulation has been reported in the induction and maintenance of cancers [35, 45]. The ERK1/2 signaling pathway is best known for its role in connecting activated growth factor receptors to changes in gene expression due to activated ERK1/2 entering the nucleus and phosphorylating transcription factors. Active ERK1/2 also translocates to a variety of other organelles including ER and mitochondria to access specific substrates and influence metabolic reprogramming during tumorigenesis [46]. Several mechanisms underlying how cellular ERK1/2 is regulated and manipulated have been elucidated, including translational regulation, scaffolding interactions, substrate competition, and crosstalk with other signaling components in each tier of the cascade [47]. It is well documented that ERK1/2 signaling can influence mitochondrial functions by regulating the expression of mitochondrial proteins in the nucleus [46, 47], but it appears that this signaling may also have intrinsic mitochondrial activities due to the mitochondrial localization of ERK1/2 [2, 48]. The present study reveals for the first time that ERK1/2 is present in the mitochondria of HNSCC cells, which is consistent with the observation in HeLa cells [49]. VDAC1 that localizes on the MOM was reported to physically associate with ERK1/2 in Hela cells [49]. In HNSCC cells, we also demonstrate that VDAC1 binds to ERK1/2 and facilitates its translocation to mitochondria, which is required for ATAD3A-ERK1/2 interaction. ERK phosphorylates an array of targets and subsequently increases the expression of several inflammatory cytokines, such as TNFα, and contributes to inflammatory responses [50]. Here, we provide evidence that TNFα is downregulated in HNSCC cells with ATAD3A loss, which implicates ATAD3A-mediated ERK1/2 signaling from the MOM towards nuclear targets. Nevertheless, further investigation is warranted into the underlying regulatory mechanisms and function of ATAD3A-ERK1/2-TNFα signaling.

Blockade of the ERK1/2 pathway represents an attractive approach for treating malignant tumors with increased ERK1/2 activity [51, 52]. As ATAD3A-mediated ERK1/2 phosphorylation is RAS-independent, it will be essential to harness this signaling together with the cytoplasmic ERK1/2 cascade to develop more effective regimens for combating cancer. Our data from animal studies have shown the therapeutic promise of this combination by the addition of salirasib treatment to the setting of ATAD3A gene suppression. Given the fact that there are no drugs currently available to inhibit ATAD3A, our study draws attention to extending the development of anti-HNSCC therapies to include novel anti-ATAD3A strategies. The promising nature of the results from this study indicates that targeting the WA motif of ATAD3A or the ATAD3A-ERK1/2 signaling node may curb the oncogenic function of ATAD3A in cancer cells. Follow-up studies in our lab include designing highly specific stapled peptides [53, 54] to incorporate a hydrophobic staple at the interface of ATAD3A-ERK1/2 proteins.

## Conclusions

Coupled with rigorous in vitro and in vivo validations, our work results in unprecedented insights into the mitochondrial oncogenic signaling mediated by ATAD3A as well as unraveling it as a promising molecular target for anti-HNSCC. Co-inhibition of ATAD3A and RAS pathways may have a better therapeutic potential for cancer treatment.

## Supplementary Information

**Additional file 1: Supplementary Figure S1.** Bioinformatic analysis reveals the clinical relevance of ATAD3A to HNSCC. **Supplementary Figure S2.** Diagram depicts that the nuclease hCas9 recruited by a sgRNA specifically recognizing a region spanning the ATAD3A codon (sgATAD3A) cleaves the ATAD3A gene. **Supplementary Figure S3.** Loss of ATAD3A induces S phase cell cycle arrest and suppresses cell invasion in HN12 cells. **Supplementary Figure S4.** Loss of ATAD3A suppresses HN8 and HN17 cell growth. **Supplementary Figure S5.** Loss of VDAC1 expression inhibits HN12 cell growth. **Supplementary Figure S6.** Knockdown of VDAC1 (shVDAC1-1) impairs the interaction between ATAD3A and ERK1/2 proteins. **Supplementary Figure S7.** 4NQO treatment upregulates ATAD3A expression in HN12 and HN8 cells. **Supplementary Figure S8.** ATAD3B is highly expressed in HNSCC cells in a ATAD3A-independent fashion.

## Data Availability

All data generated or analyzed during this study are included in this published article [and its supplementary information files].

## Abbreviations

3D: Three-dimensional
ATAD3A: The ATPase family AAA-domain containing protein 3A
Cas9n: The D10A nickase mutant of Cas9
DEGs: Differentially expressed genes
ER: Endoplasmic reticulum
GO: Gene Ontology
HNSCC: Head and neck squamous cell carcinoma
NSG: NOD.Cg-*Prkdcscid Il2rgtm1Wjl/SzJ*
IF: Immunofluorescence
IP: Immunoprecipitation
IHC: Immunohistochemistry
MIM: Mitochondrial inner membrane
MOM: Mitochondrial outer membrane
OXPHOS: Oxidative phosphorylation
RNA-seq: RNA-sequencing
RT-qPCR: Reverse transcription quantitative PCR
sgRNA: Single guided RNA
shRNA: Short hairpin RNA
TEM: Transmission electron microscopy
WA: Walker A
WASF3: The WAS protein family member 3
WB: Walker B

## References

[1] Dickerson T, Jauregui CE, Teng Y (2017). Friend or foe? Mitochondria as a pharmacological target in cancer treatment. Future Med Chem.

[2] Vyas S, Zaganjor E, Haigis MC (2016). Mitochondria and cancer. Cell.

[3] Teng Y, Ren X, Li H, Shull A, Kim J, Cowell JK (2016). Mitochondrial ATAD3A combines with GRP78 to regulate the WASF3 metastasis-promoting protein. Oncogene.

[4] Peralta S, Goffart S, Williams SL, Diaz F, Garcia S, Nissanka N (2018). ATAD3 controls mitochondrial cristae structure in mouse muscle, influencing mtDNA replication and cholesterol levels. J Cell Sci.

[5] Lang L, Loveless R, Teng Y (2020). Emerging links between control of mitochondrial protein ATAD3A and Cancer. Int J Mol Sci.

[6] Cooper HM, Yang Y, Ylikallio E, Khairullin R, Woldegebriel R, Lin KL (2017). ATPase-deficient mitochondrial inner membrane protein ATAD3A disturbs mitochondrial dynamics in dominant hereditary spastic paraplegia. Hum Mol Genet.

[7] Gilquin B, Taillebourg E, Cherradi N, Hubstenberger A, Gay O, Merle N (2010). The AAA+ ATPase ATAD3A controls mitochondrial dynamics at the interface of the inner and outer membranes. Mol Cell Biol.

[8] Teng Y, Lang L, Shay C (2019). ATAD3A on the path to Cancer. Adv Exp Med Biol.

[9] Baudier J (2018). ATAD3 proteins: brokers of a mitochondria-endoplasmic reticulum connection in mammalian cells. Biol Rev Camb Philos Soc.

[10] Jin G, Xu C, Zhang X, Long J, Rezaeian AH, Liu C (2018). Atad3a suppresses Pink1-dependent mitophagy to maintain homeostasis of hematopoietic progenitor cells. Nat Immunol.

[11] Peralta S, González-Quintana A, Ybarra M, Delmiro A, Pérez-Pérez R, Docampo J (2019). Novel ATAD3A recessive mutation associated to fatal cerebellar hypoplasia with multiorgan involvement and mitochondrial structural abnormalities. Mol Genet Metab.

[12] Fang HY, Chang CL, Hsu SH, Huang CY, Chiang SF, Chiou SH (2010). ATPase family AAA domain-containing 3A is a novel anti-apoptotic factor in lung adenocarcinoma cells. J Cell Sci.

[13] Huang KH, Chow KC, Chang HW, Lin TY, Lee MC (2011). ATPase family AAA domain containing 3A is an anti-apoptotic factor and a secretion regulator of PSA in prostate cancer. Int J Mol Med.

[14] Gao L, Zhao X, Lang L, Shay C, Yeudall WA, Teng Y (2018). Autophagy blockade sensitizes human head and neck squamous cell carcinoma towards CYT997 through enhancing excessively high reactive oxygen species-induced apoptosis. J Exp Clin Cancer Res.

[15] Farwell DG, Shera KA, Koop JI, Bonnet GA, Matthews CP, Reuther GW (2000). Genetic and epigenetic changes in human epithelial cells immortalized by telomerase. Am J Pathol.

[16] Gao L, Lang L, Zhao X, Shay C, Shull AY, Teng Y (2019). FGF19 amplification reveals an oncogenic dependency upon autocrine FGF19/FGFR4 signaling in head and neck squamous cell carcinoma. Oncogene..

[17] Camara AK, Zhou Y, Wen PC, Tajkhorshid E, Kwok WM (2017). Mitochondrial VDAC1: a key gatekeeper as potential therapeutic target. Front Physiol.

[18] Zhao X, Lang L, He L, Gao L, Chyan D, Xiong Y (2019). Intracellular reduction in ATP levels contributes to CYT997-induced suppression of metastasis of head and neck squamous carcinoma. Cell Mol Med.

[19] Holt IJ, He J, Mao CC, Boyd-Kirkup JD, Martinsson P, Sembongi H (2007). Mammalian mitochondrial nucleoids: organizing an independently minded genome. Mitochondrion.

[20] Hubstenberger A, Merle N, Charton R, Brandolin G, Rousseau D (2010). Topological analysis of ATAD3A insertion in purified human mitochondria. J Bioenerg Biomembr.

[21] Ran FA, Hsu PD, Wright J, Agarwala V, Scott DA, Zhang F (2013). Genome engineering using the CRISPR-Cas9 system. Nat Protoc.

[22] Teng Y, Ngoka L, Mei Y, Lesoon L, Cowell JK (2012). HSP90 and HSP70 proteins are essential for stabilization and activation of WASF3 metastasis-promoting protein. J Biol Chem.

[23] Lang L, Lam T, Chen A, Jensen C, Duncan L, Kong FC (2020). Circumventing AKT-associated Radioresistance in oral cancer by novel nanoparticle-encapsulated capivasertib. Cells.

[24] Sun FC, Wei S, Li CW, Chang YS, Chao CC, Lai YK (2006). Localization of GRP78 to mitochondria under the unfolded protein response. Biochem J.

[25] He L, Gao L, Shay C, Lang L, Lv F, Teng Y (2019). Histone deacetylase inhibitors suppress aggressiveness of head and neck squamous cell carcinoma via histone acetylation-independent blockade of the EGFR-Arf1 axis. J Exp Clin Cancer Res.

[26] Teng Y, Qin H, Bahassan A, Bendzunas NG, Kennedy EJ, Cowell JK (2016). The WASF3-NCKAP1-CYFIP1 complex is essential for breast cancer metastasis. Cancer Res.

[27] Lang L, Shay C, Zhao X, Teng Y (2017). Combined targeting of Arf1 and Ras potentiates anticancer activity for prostate cancer therapeutics. J Exp Clin Cancer Res.

[28] Lang L, Wang F, Ding Z, Zhao X, Loveless R, Xie J (2021). Blockade of glutamine-dependent cell survival augments antitumor efficacy of CPI-613 in head and neck cancer. J Exp Clin Cancer Res.

[29] Nicholas D, Proctor EA, Raval FM, Ip BC, Habib C, Ritou E (2017). Advances in the quantification of mitochondrial function in primary human immune cells through extracellular flux analysis. PLoS One.

[30] Lang L, Shay C, Zhao X, Xiong Y, Wang X, Teng Y (2019). Simultaneously inactivating Src and AKT by saracatinib/capivasertib co-delivery nanoparticles to improve the efficacy of anti-Src therapy in head and neck squamous cell carcinoma. J Hematol Oncol.

[31] Xiong Y, He L, Shay C, Lang L, Loveless J, Yu J (2019). Nck-associated protein 1 associates with HSP90 to drive metastasis in human non-small-cell lung cancer. J Exp Clin Cancer Res.

[32] Jensen C, Teng Y (2020). Is it time to start transitioning from 2D to 3D cell culture?. Front Mol Biosci.

[33] Rone MB, Midzak AS, Issop L, Rammouz G, Jagannathan S, Fan J, Papadopoulos V (2012). Identification of a dynamic mitochondrial protein complex driving cholesterol import, trafficking, and metabolism to steroid hormones. Mol Endocrinol.

[34] Arif T, Vasilkovsky L, Refaely Y, Konson A, Shoshan-Barmatz V (2014). Silencing VDAC1 expression by siRNA inhibits cancer cell proliferation and tumor growth in vivo. Mol Ther Nucleic Acids.

[35] Downward J (2003). Targeting RAS signalling pathways in cancer therapy. Nat Rev Cancer.

[36] Siegel RL, Miller KD, Jemal A (2015). Cancer statistics, 2015. CA Cancer J Clin.

[37] Miranda-Galvis M, Loveless R, Kowalski LP, Teng Y (2021). Impacts of environmental factors on head and neck cancer pathogenesis and progression. Cells.

[38] Shu L, Hu C, Xu M, Yu J, He H, Lin J (2021). ATAD3B is a mitophagy receptor mediating clearance of oxidative stress-induced damaged mitochondrial DNA. EMBO J.

[39] Desai R, Frazier AE, Durigon R, Patel H, Jones AW, Dalla Rosa I (2017). ATAD3 gene cluster deletions cause cerebellar dysfunction associated with altered mitochondrial DNA and cholesterol metabolism. Brain.

[40] Merle N, Féraud O, Gilquin B, Hubstenberger A, Kieffer-Jacquinot S, Assard N (2012). ATAD3B is a human embryonic stem cell specific mitochondrial protein, re-expressed in cancer cells, that functions as dominant negative for the ubiquitous ATAD3A. Mitochondrion.

[41] Costantini P, Jacotot E, Decaudin D, Kroemer G (2000). Mitochondrion as a novel target of anticancer chemotherapy. J Natl Cancer Inst.

[42] Wallace DC (2012). Mitochondria and cancer. Nat Rev Cancer.

[43] Gilquin B, Cannon BR, Hubstenberger A, Moulouel B, Falk E, Merle N (2010). The calcium-dependent interaction between S100B and the mitochondrial AAA ATPase ATAD3A and the role of this complex in the cytoplasmic processing of ATAD3A. Mol Cell Biol.

[44] Harel T, Yoon WH, Garone C, Gu S, Coban-Akdemir Z, Eldomery MK (2016). Recurrent de novo and biallelic variation of ATAD3A, encoding a mitochondrial membrane protein, results in distinct neurological syndromes. Am J Hum Genet.

[45] Plotnikov A, Flores K, Maik-Rachline G, Zehorai E, Kapri-Pardes E, Berti DA (2015). The nuclear translocation of ERK1/2 as an anticancer target. Nat Commun.

[46] Papa S, Choy PM, Bubici C (2019). The ERK and JNK pathways in the regulation of metabolic reprogramming. Oncogene.

[47] Wortzel I, Seger R (2011). The ERK cascade: distinct functions within various subcellular organelles. Genes Cancer.

[48] Baines CP, Zhang J, Wang GW, Zheng YT, Xiu JX, Cardwell EM (2002). Mitochondrial PKCε and MAPK form signaling modules in the murine heart: enhanced mitochondrial PKCε-MAPK interactions and differential MAPK activation in PKCε-induced cardioprotection. Circ Res.

[49] Galli S, Jahn O, Hitt R, Hesse D, Opitz L, Plessmann U (2009). A new paradigm for MAPK: structural interactions of hERK1 with mitochondria in HeLa cells. PLoS One.

[50] Sabio G, Davis RJ (2014). TNF and MAP kinase signalling pathways. Semin Immunol.

[51] Judd NP, Winkler AE, Murillo-Sauca O, Brotman JJ, Law JH, Lewis JS (2012). ERK1/2 regulation of CD44 modulates oral cancer aggressiveness. Cancer Res.

[52] Davis JE, Xie X, Guo J, Huang W, Chu WM, Huang S (2016). ARF1 promotes prostate tumorigenesis via targeting oncogenic MAPK signaling. Oncotarget.

[53] Teng Y, Bahassan A, Dong D, Hanold LE, Ren X, Kennedy EJ (2016). Targeting the WASF3-CYFIP1 complex using stapled peptides suppresses cancer cell invasion. Cancer Res.

[54] Xie X, Gao L, Shull AY, Teng Y (2016). Stapled peptides: providing the best of both worlds in drug development. Future Med Chem.

