# Supplementary material for: ATAD3A mediates activation of RAS-independent mitochondrial ERK1/2 signaling, favoring head and neck cancer development
Source: J Exp Clin Cancer Res. 2022 Jan 29;41:43. doi: 10.1186/s13046-022-02274-9 (PMC8800319; doi:10.1186/s13046-022-02274-9)
Supplement: Supplementary file 1 — Additional file 1: Supplementary Figure S1. Bioinformatic analysis reveals the clinical relevance of ATAD3A to HNSCC. Supplementary Figure S2. Diagram depicts that the nuclease hCas9 recruited by a sgRNA specifically recognizing a region spanning the ATAD3A codon (sgATAD3A) cleaves the ATAD3A gene. Supplementary Figure S3. Loss of ATAD3A induces S phase cell cycle arrest and suppresses cell invasion in HN12 cells. Supplementary Figure S4. Loss of ATAD3A suppresses HN8 and HN17 cell growth. Supplementary Figure S5. Loss of VDAC1 expression inhibits HN12 cell growth. Supplementary Figure S6. Knockdown of VDAC1 (shVDAC1-1) impairs the interaction between ATAD3A and ERK1/2 proteins. Supplementary Figure S7. 4NQO treatment upregulates ATAD3A expression in HN12 and HN8 cells. Supplementary Figure S8. ATAD3B is highly expressed in HNSCC cells in a ATAD3A-independent fashion. [file 13046_2022_2274_MOESM1_ESM.docx]

**Supplemental Information for**

**ATAD3A mediates activation of RAS-independent mitochondrial ERK1/2 signaling, favoring head and neck cancer development**

Liwei Lang^1^, Reid Loveless^1^, Juan Dou^2^, Tiffany Lam^1^, Alex Chen^1^, Fang Wang^1^, Li Sun^1^, Jakeline Juarez^1^, Zhaohui Steve Qin^3^, Nabil F Saba^2^, Chloe Shay^4^, Yong Teng^1,2*^

**Correspondence:** Yong Teng, [yong.teng@emory.edu](mailto:yong.teng@emory.edu)

**This PDF file includes:**

**Supplementary Figures and Figure legends**

**Supplementary Figure S1. Bioinformatic analysis reveals the clinical relevance of ATAD3A to HNSCC.** (A) The gene expression of ATAD3A in different cancers based Oncomine database. (B) The expression of ATAD3A in different tumor stages of HNSCC based on TCGA data.


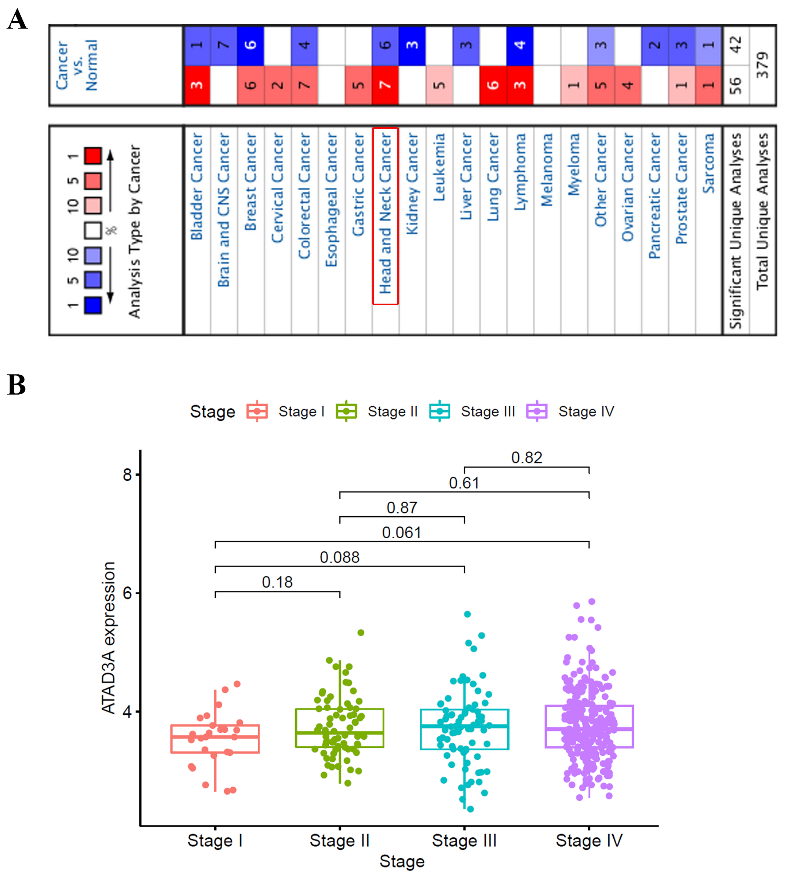


**Supplementary Figure S2. Diagram depicts that the nuclease hCas9 recruited by a sgRNA specifically recognizing a region spanning the ATAD3A codon (sgATAD3A) cleaves the ATAD3A gene.** The vertical arrow shows the cleavage site.

**
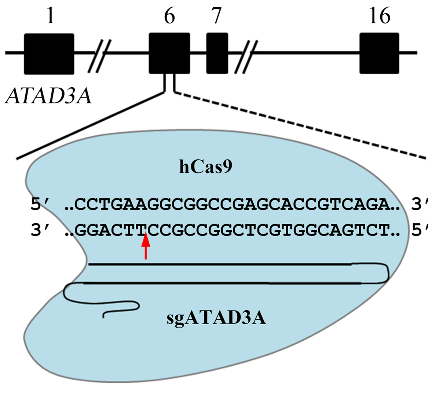
**

**Supplementary Figure S3. Loss of ATAD3A induces S phase cell cycle arrest and suppresses cell invasion in HN12 cells.** (A) The effect of ATAD3A KO (#1 and #5) on cell cycle determined by flow cytometry after PI staining. Quantitative data from flow cytometry are shown in the right panel (n=3). (B) The effect of ATAD3A KO on cell invasion determined by Matrigel transwell. Quantitative data from invasion assays are shown in the right panel (n=3). (C) The effect of ATAD3A KO on cell cycle, apoptosis and invasion-related molecules. **p*<0.05; ***p*<0.01.

**
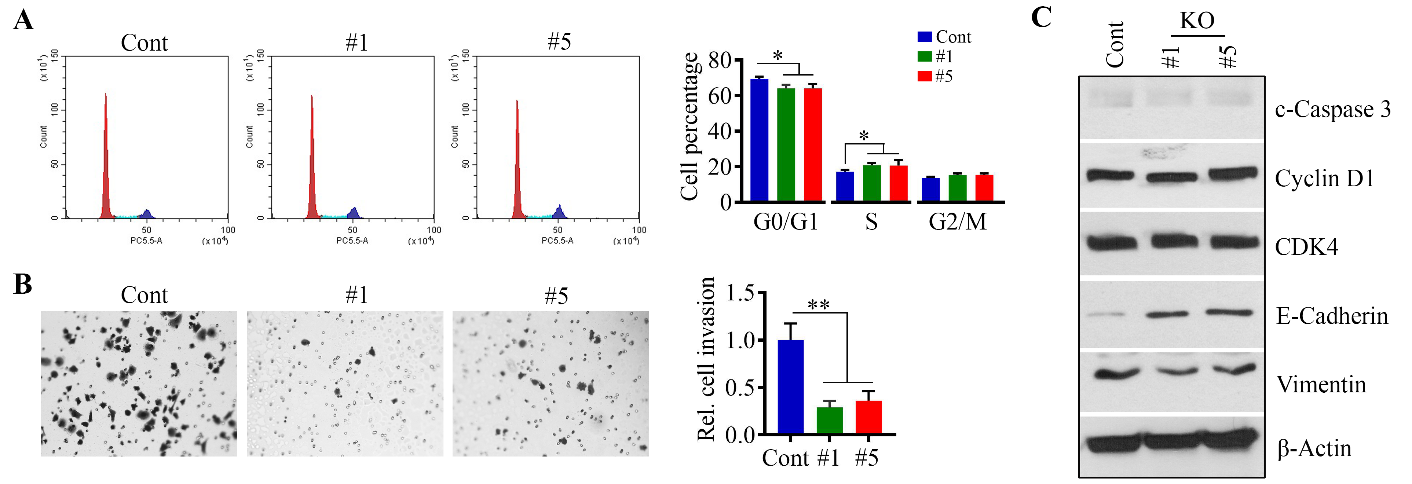
**

**Supplementary Figure S4. Loss of ATAD3A suppresses HN8 and HN17 cell growth.** (A) The knockdown effect of shRNAs against ATAD3A (sh3A-1 and sh3A-2). (B) The effect of ATAD3A knockdown on cell proliferation on Day 3. (C) The effect of ATAD3A knockout on cell colony formation within 3 weeks. Quantitative data from colony formation assays are shown in the right panel (n=3). **p*<0.05.


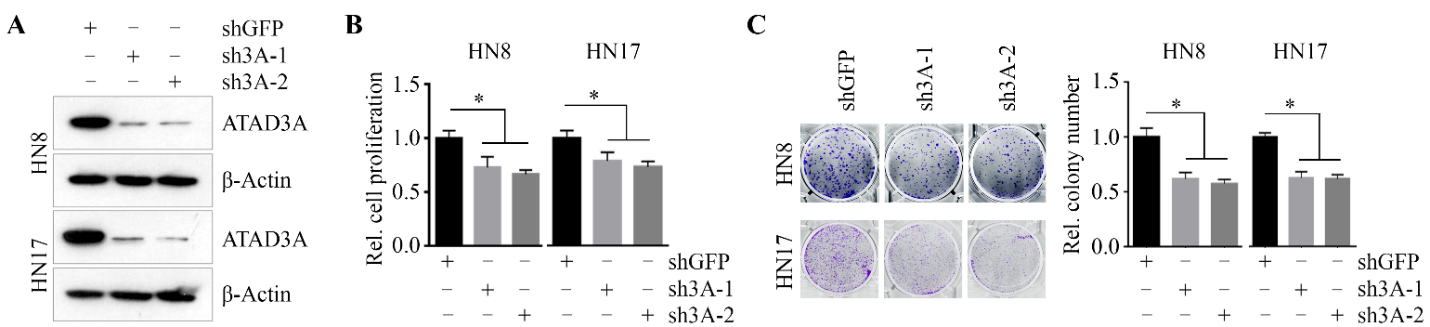


**Supplementary Figure S5. Loss of VDAC1 expression inhibits HN12 cell growth.** (A) The effect of VDAC1 knockdown (shVDAC1-1, shVDAC1-2) on cell proliferation on Day 3. (B) The effect of VDAC1 knockdown on colony formation within 3 weeks. Quantitative data from colony formation assays are shown in the right panel (n=3). **p*<0.05; ***p*<0.01.

**
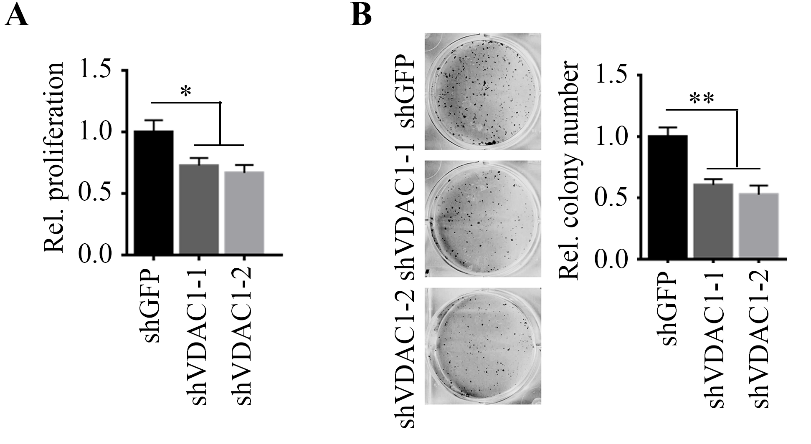
**

**Supplementary Figure S6. Knockdown of VDAC1 (shVDAC1-1) impairs the interaction between ATAD3A and ERK1/2 proteins.** The immunoprecipitates from HN12 cells were pulled down using anti-ATAD3A antibody**.**


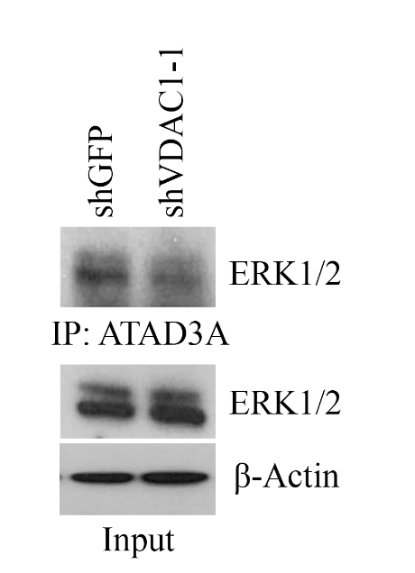


**Supplementary Figure S7. 4NQO treatment upregulates ATAD3A expression in HN12 and HN8 cells.**

**
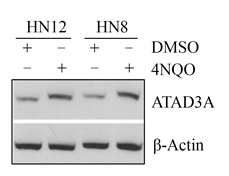
**

**Supplementary Figure S8. ATAD3B is highly expressed in HNSCC cells in a ATAD3A-independent fashion.** (A) ATAD3B mRNA levels in various HNSCC cell lines and normal oral keratinocytes (hTERT). (B) ATAD3B mRNA levels in ATAD3A KO HN12 cells. Quantitative data from RT-qPCR assays are shown (n=3). ***p*<0.01.

**
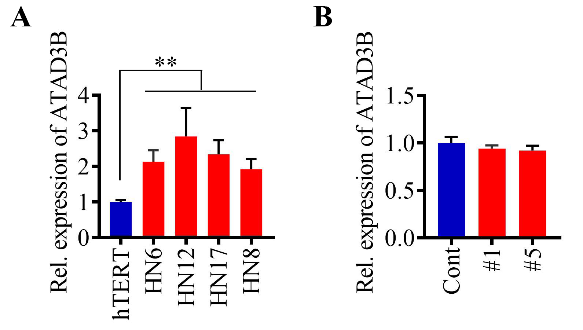
**
